# Nonpharmacological Treatment of Rhinoconjunctivitis and Rhinosinusitis

**DOI:** 10.1155/2014/416236

**Published:** 2014-12-21

**Authors:** Ralph Mösges, Carlos E. Baena-Cagnani, Desiderio Passali

**Affiliations:** ^1^Institute of Medical Statistics, Informatics and Epidemiology (IMSIE), University Hospital of Cologne, Cologne, Germany; ^2^Postgraduate Department of the Faculty of Medicine, Catholic University of Cordoba, Cordoba, Argentina; ^3^University of Siena, ENT Unit, Siena, Italy

Infectious and allergic diseases of the upper airways are among the most common illnesses of all age groups. Numerous guidelines have been issued for the evidence-based treatment of these diseases. Therapy involves local or systemic application of well-characterized pharmacologically active medications such as glucocorticosteroids, antihistamines, leukotriene-receptor antagonists, alpha-adrenergic receptor agonists, mast-cell stabilizers, and a monoclonal antibody targeting specific immunoglobulin E. In spite of the fact that the various therapeutic options have proven their efficacy and effectiveness in a myriad of well-designed clinical trials, many patients express their discontent with current therapies. In several surveys a majority of patients stated that they would prefer nonpharmacological “nonchemical” treatment over what is currently prescribed or recommended by their physicians. This desire stands in clear contrast to a systematic review conducted some years ago proving that there was practically no evidence at that time for the efficacy of alternative forms of treatment of allergic diseases of the upper airways.

Ever since, several meta-analyses, systematic reviews, and well-designed clinical trials have been published, bearing witness to the scientific basis of some nonpharmacologic options like nasal irrigation in the treatment of different pathologies of the upper airways, namely, for rhinosinusitis and for allergic rhinoconjunctivitis.

In this special issue, we want to highlight some new approaches that until lately have found less public attention in this domain.

In their article on the clinical efficacy of a spray containing hyaluronic acid and dexpanthenol after surgery in the nasal cavity, I. Gouteva et al. demonstrate beneficial effects on wound healing for two substances that have a long history of local application in the nose but formerly were used separately.

The so-called “extremolyte” ectoine is a substance, which was introduced a few years ago as a “natural” treatment of allergic and inflammatory pathologies of the skin and the mucosal tissues. Five articles in this issue illuminate the mechanism of action and the potential benefits of a nasal spray containing ectoine in diseases of various aetiologies like rhinitis sicca or allergic rhinitis.

A form of local treatment of the nasal mucosa that is proximate to ectoine is the nasal spray containing liposomes. Its use in allergic rhinitis has been well established. A. Eitenmüller and coauthors present in their article for the first time data on ectoine in chronic rhinosinusitis.

For patients who are sceptical about using any sort of active ingredients in their nasal spray, the application of mere water in the form of thermal water inhalations could be an alternative. S. Keller et al. have conducted a meta-analysis which demonstrates some benefits of this least-invasive local therapy.

There has been much controversy over the last decade on the meaningfulness of probiotics in preventing allergies. New forms now come as treatment of chronic diseases of the upper airways. M. F. Kramer and his coauthor M. D. Heath present the latest state of knowledge of this nutritional therapeutic approach.

Another much debated therapy in the field of allergic diseases is acupuncture. Most investigations have previously focussed on pollen-allergic patients. It is the merit of Hauswald and coauthors to have conducted a randomized controlled clinical trial using acupuncture as an alternative to treatment with the state-of-the-art antihistamine loratadine in house dust allergic patients.

It has been the intention of the editors of this special issue to round up our knowledge on alternative therapies for upper airways diseases beyond the fields of herbal medicine and homeopathy. The number of articles that have been submitted but more so their quality was a positive surprise.

We are very grateful to the publishers who have had the courage of disseminating unusual ideas. Ghada Ali and Marie-Josefine Joisten were the driving spirits behind the scenes and without their continuous commitment this special issue would not have seen the light of the day. We are deeply obliged and thankful for their endeavours.

*Ralph Mösges* *Ralph Mösges* *Carlos E. Baena-Cagnani* *Carlos E. Baena-Cagnani* *Desiderio Passali* *Desiderio Passali*

